# A Novel Drug–Disease Association Prediction Method Based on Deep Non-Negative Matrix Factorization with Local Graph Feature

**DOI:** 10.1007/s12539-025-00733-3

**Published:** 2025-07-07

**Authors:** Mengyun Yang, Bin Yang, Jiajun Chen, Xiwei Tang, Guihua Duan

**Affiliations:** 1 School of Computer Science, Hunan First Normal University, Changsha, 410205 China; 2 School of Mechanical and Energy Engineering, Shaoyang University, Shaoyang, 422000 China; 3 School of Computer Science and Engineering, Central South University, Changsha, 410000 China

**Keywords:** Drug repositioning, Multi-similarity, Deep non-negative matrix factorization

## Abstract

**Abstract:**

Computational drug repurposing utilizes data analysis and predictive models to identify new uses for existing drugs and new drugs, significantly improving research efficiency and reducing costs compared to traditional screening methods. Due to the limitations of current computational models in extracting deep key features, we develop a novel drug repurposing model based on the deep non-negative matrix factorization (DNMF-DDA) to enhance the accuracy of drug-disease association predictions. The model leverages similarity and known association data to extract low-rank features from complex data spaces, allowing for the prediction of potential drug-disease associations. To improve performance for novel drugs, we apply the *k*-nearest neighbors (KNN) algorithm for preprocessing, increasing the density of the matrix’s prior information. Next, we construct two integrated matrices based on the similarities of drugs and diseases, respectively, and the optimized association data. During deep matrix factorization, we incorporate graph Laplacian and relaxed regularization constraints to optimize local graph features. This multi-layer optimization enhances the model’s understanding of complex drug-disease relationships, effectively mitigating the negative impact of insufficient prior information during cold-start tests. Furthermore, we incorporate non-negativity constraints to ensure that the prediction results are biologically meaningful. To evaluate the performance of DNMF-DDA, we conducted cold-start test and 10-fold cross-validation on three datasets and systematically compared it with five state-of-the-art drug repurposing methods. The results demonstrate that DNMF-DDA performs exceptionally well in predicting drug-disease associations, significantly outperforming existing approaches. Our proposed method not only efficiently handles high-dimensional data but also exhibits superior performance, providing new insights for drug development. Moreover, the case study further validated the significant practical value of the DNMF-DDA model in practical applications.

**Graphical Abstract:**

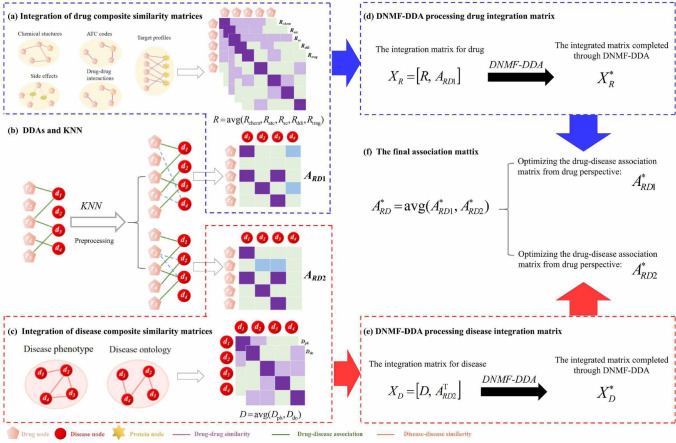

## Introduction

Despite substantial financial investments in the global drug sector, market approvals for new medications have remained scarce in recent decades [1]. This is largely attributed to the lengthy process of testing therapeutic compounds, which typically spans 10 to 15 years before a drug can be approved for commercial use. To address this challenge and accelerate drug discovery, repurposing already commercialized drugs is increasingly attracting the interest of biomedical researchers and pharmaceutical companies [2].

Optimizing the use of existing drugs for alternative therapeutic purposes can significantly lower development costs while maintaining safety. In recent years, several drugs have been successfully repurposed through clinical trials, saving pharmaceutical companies significant development costs while generating substantial economic value [3]. For instance, clemastine, originally used as an antihistamine, has been repurposed as an antiviral drug [4]; sertraline, initially developed as an antidepressant, has found new use as an antifungal agent [5]; disulfiram, formerly used to treat alcoholism, has been repurposed as an antimicrobial drug [6, 7]. Although manual and theoretical research has successfully repurposed certain drugs, this approach remains time-consuming and difficult to scale. Therefore, systematic computational drug repurposing has emerged as a crucial direction in the life sciences, leveraging advanced algorithmic models to efficiently identify potential drug-disease associations (DDAs) and accelerate the repurposing process.

In recent years, various computational approaches have emerged to predict possible DDAs, offering new avenues for drug repurposing. Among these, methods based on heterogeneous biological networks have proven particularly valuable. These approaches construct homogeneous or heterogeneous networks to infer biological similarities and use computational techniques to extract feature information from the network nodes for DDA prediction [8]. For instance, Wang et al. [9] introduced the TL-HGBI method, which integrates multi-omics information from diseases, drugs, and targets via a three-layer heterogeneous network to predict DDAs. Luo et al. [10] introduced the MBiRW model, which combines similarity metrics and a bidirectional random walk algorithm to learn feature information from drug-disease heterogeneous networks, enabling the identification of potential drug indications. To further integrate multi-source data and expand the application of random walk models in drug-disease-target heterogeneous networks, Luo et al. [11] subsequently proposed a novel model, RWHNDR, which leverages global multi-source information from heterogeneous networks to enhance predictive performance.

As biomedical research advances, new data sources continue to emerge, making it essential to learn complex patterns from diverse datasets. Machine learning methods play a critical role in predicting DDAs [12]. Napolitano et al. [13] proposed a multiclass support vector machine (SVM) classifier that integrates multi-omics data from drugs to facilitate drug repurposing. Similarly, Oh et al. [14] designed a random forest-based classifier that quantifies the topological characteristics of drug-disease spaces to predict potential indications within integrated genetic networks. However, traditional machine learning methods rely on feature selection to build predictive models, which can suffer from issues like small datasets and high noise, reducing reliability. In contrast, deep learning, by employing multi-layer neural networks, learns higher-level abstract features from data and uncovers underlying structures, enabling the discovery of patterns in large datasets and improving prediction accuracy [15]. In recent years, deep learning and graph learning have advanced significantly in bioinformatics [16, 17]. Lin et al. introduced the GraphCPI framework, which integrates molecular graph topology with protein sequence chemical information [18]. By leveraging GNNs for compound feature modeling and employing Prot2Vec and CNNs for protein semantic extraction, GraphCPI overcomes the constraints of conventional molecular docking, improving both the precision and applicability of compound-protein interaction prediction. Lei et al. [19] introduced a novel drug repurposing method, DRDSA, which utilizes a deep sparse autoencoder to learn the nonlinear structures within drug-disease feature networks, generating low-dimensional feature representations and inferring potential DDAs. Furthermore, Liu et al. [20] proposed the structure-enhanced graph convolutional network (SLGCN), which optimizes message aggregation via a relational matrix and combines it with graph convolutional networks to deeply learn node features, significantly improving the accuracy of DDA predictions in heterogeneous networks.

Biomedical data are often highly sparse and involve complex DDAs. To enhance the scalability and interpretability of drug repurposing methods, matrix factorization techniques have played a crucial role in this field. Specifically, these approaches capture the latent features of DDAs through low-rank matrices and leverage prior knowledge to fill in missing associations in sparse datasets [21]. Zhang et al. [22] proposed the SCMFDD method, which projects DDAs into a low-dimensional space using prior information and employs matrix factorization and similarity constraints to uncover latent features and predict unknown DDAs. Yang et al. [23] introduced the bounded nuclear norm regularization (BNNR) method, which derives the optimal correlation matrix under low-order assumptions by employing a matrix-filling approach. This method ensures biologically meaningful predictions while efficiently handling highly noisy data. Additionally, Yang et al. [24] proposed the MSBMF method, which dynamically integrates multi-omics data to optimize the association matrix and predict potential DDAs. Notably, extracting key feature information from heterogeneous biological networks has emerged as a pivotal challenge in drug repurposing research.

**Fig. 1:**
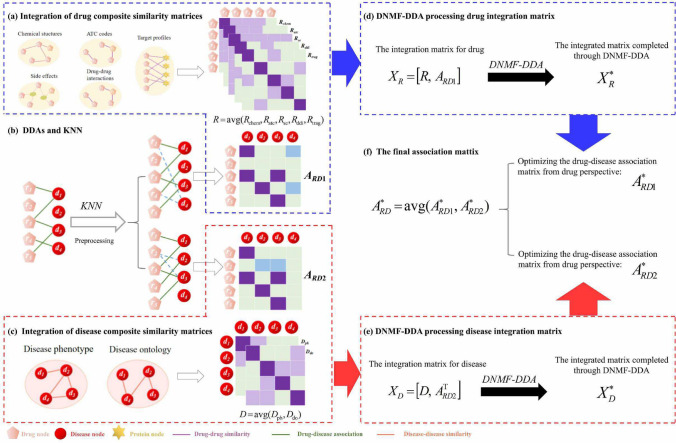
The overall workflow of DNMF-DDA. **a** Integration of drug composite similarity matrices. **b** DDAs and KNN. **c** Integration of disease composite similarity matrices. **d** DNMF-DDA processing drug integration matrix. **e** DNMF-DDA processing disease integration matrix. **f** The final association matrix

This study introduces an advanced drug repurposing model leveraging deep non-negative matrix factorization (DNMF-DDA). It can extract effective low-dimensional feature representations in high-dimensional complex data spaces and leverage low-rank prior information for predicting potential DDAs. The overall workflow of the DNMF-DDA model is illustrated in Fig. 1. The key contributions of this model are as follows:

- We propose an effective method, DNMF-DDA, for predicting DDAs based on the deep non-negative matrix factorization with local graph features. It can deeply mine data relationships and capture local structural information.
- DNMF-DDA utilizes the graph Laplacian operator and relaxed regularization constraints. The former analyzes graph-based data structure, and the latter maintains the consistency of the matrix hierarchical structure. This combination obtains more accurate local potential representations.
- A layer-wise iterative strategy is proposed to ensure the efficient convergence of the DNMF-DDA model.

## Materials

In evaluating the proposed model’s effectiveness and generalization capability, we used Gdataset [25], Cdataset [10], and our newly collected dataset (CTDdataset2023). The information for these datasets is sourced from DrugBank [26], the OMIM database [27], and the CTD database [28]. The specific numbers of drugs, diseases, and known associations for each dataset are detailed in Table 1. Notably, CTDdataset2023 was constructed by integrating the latest data from the CTD database. DDAs are represented by a binary matrix ${{\varvec{A}}} \in \mathbb {R}^{m \times n}$, where 1 indicates a known association and 0 indicates an unknown one. To fully explore the potential links between drugs and diseases and extract additional key features, we employed various similarity metrics.

For drugs, the chemical structure similarity (${{\varvec{R}}}_{\textrm{c h e m}}$) measures the structural similarity of drug compounds and is computed using the chemical development kit (CDK) [29]. ATC code similarity ${{\varvec{R}}}_{\textrm{a t c}}$ is derived from semantic similarity algorithms that calculate the similarity between ATC codes. The drug-drug interaction similarity (${{\varvec{R}}}_{\textrm{d d i}}$) and the target profile similarity (${{\varvec{R}}}_{\textrm{t a r g}}$) are extracted from DrugBank and their similarity scores are calculated using the Jaccard index [30]. The side effect similarity (${{\varvec{R}}}_{\textrm{s e}}$) is sourced from the SIDER database [31] and calculated using the Jaccard similarity coefficient to compare side effect profiles. For diseases, the phenotype similarity (${{\varvec{D}}}_{\textrm{p h}}$) data is obtained from MimMiner [32], while the disease ontology similarity (${{\varvec{D}}}_{\textrm{d o}}$) is computed based on the structure of gene ontology [33].

Finally, by averaging the similarity matrices, we constructed the comprehensive drug similarity matrix (${{\varvec{R}}} \in \mathbb {R}^{m \times m}$) and the comprehensive disease similarity matrix (${{\varvec{D}}} \in \mathbb {R}^{n \times n}$) for further analysis. These similarity metrics serve as key prior information to support the prediction of potential DDAs.

**Table 1:** The count of drugs, diseases, and corresponding associations in three datasets

Dataset	Drugs	Diseases	Association pairs	Sparsity
$\text {Gdataset}^{[1]}$	593	313	1,933	1.04%
$\text {Cdataset}^{[2]}$	663	409	2,352	0.87%
CTDdataset2023	1,327	278	3,740	1.01%

## Methods

This section presents an overview of the drug repurposing model based on deep non-negative matrix factorization. We first introduce the principles of the basic non-negative matrix factorization (BasicNMF) algorithm [34] and the basic deep non-negative matrix factorization (BDNMF) algorithm [35]. Building on these, we introduce the DNMF-DDA model to predict unknown indications of drugs. Finally, we demonstrate the detailed solution process of DNMF-DDA.

### Basic Non-negative Matrix Factorization (BasicNMF)

NMF is an efficient data analysis method commonly used in areas like image classification [36], text mining [37], and user preference prediction [38]. The core idea is to approximate the original matrix using multiplicative update rules, decomposing the non-negative matrix ***X*** into two low-rank non-negative matrices ***W*** and ***H***, such that ${{\varvec{X}}} \approx {{\varvec{W}}} {{\varvec{H}}}$. Its objective function is generally expressed as

1 $$\begin{aligned} \begin{aligned} \min _{{{\varvec{W}}}, {{\varvec{H}}}}\left\| {{\varvec{X}}}-{{\varvec{W}}} {{\varvec{H}}}\right\| _\textrm{F}^2 \quad \text{ s.t. } \quad {{\varvec{W}}} \ge 0 {{\varvec{H}}} \ge 0 \end{aligned} \end{aligned}$$

Where ***W*** represents the basis matrix, and ***H*** denotes the weight coefficient matrix for the basis vectors. In the context of drug repurposing, the application of NMF allows for the decomposition of known drug activity data, extracting features that reflect underlying mechanisms, and reorganizing the information to reveal potential activity patterns between drugs and various diseases.

### Basic Deep Non-Negative Matrix Factorization (BDNMF)

**Fig. 2:**
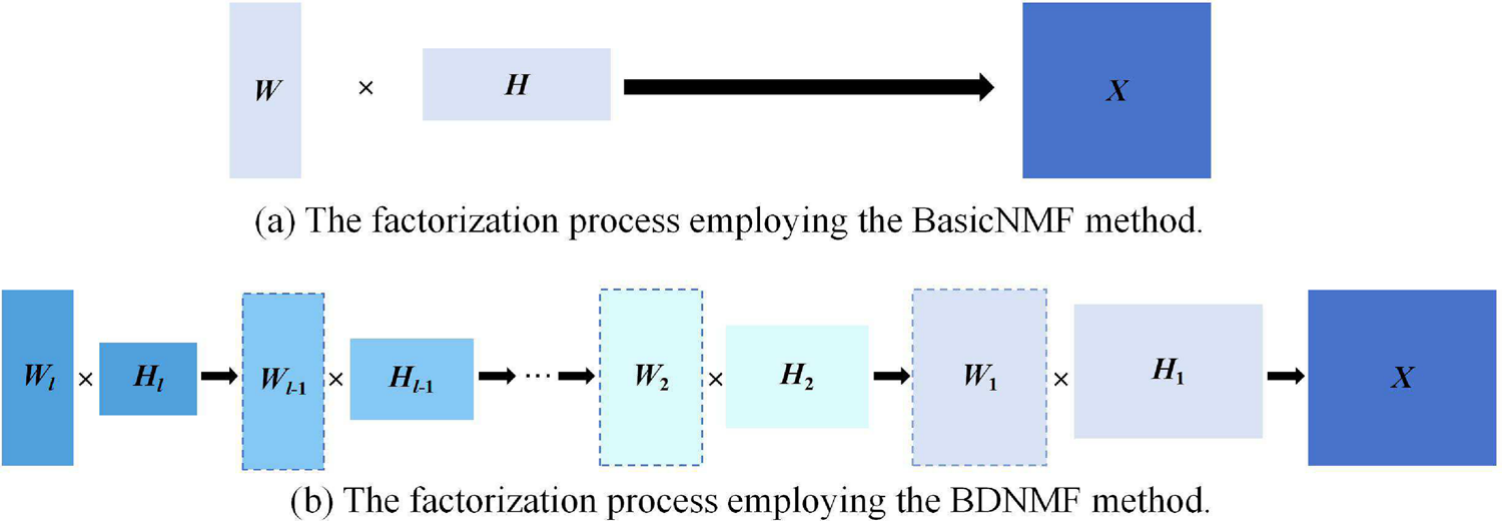
Matrix factorization process using the BasicNMF algorithm and the BDNMF algorithm

While traditional NMF and its variants demonstrate some capability in feature extraction, shallow factorization fails to uncover more complex data patterns [39]. In contrast, deep architectures have shown significant advantages in the fields of image processing and analysis [40, 41] (Fig. 2).

The BDNMF method uncovers deeper structures within the data through multi-level decomposition, capturing the essential features at higher levels of abstraction [35]. Compared to traditional shallow decomposition techniques, this method demonstrates superior performance in data representation. The BDNMF model builds a multi-layered structure on the basis matrix, allowing for multi-layer factorization to effectively capture the deeper features within the dataset. Specifically, the design of BDNMF consists of *l* layers, with each layer progressively abstracting the input data to reveal more intrinsic data characteristics.

2 $$\begin{aligned} \left\{ \begin{aligned}&{{\varvec{X}}}={{\varvec{W}}}_1 {{\varvec{H}}}_1 \\&{{\varvec{W}}}_1={{\varvec{W}}}_2 {{\varvec{H}}}_2 \\&\ldots \\&{{\varvec{W}}}_{l-1}={{\varvec{W}}}_l {{\varvec{H}}}_l \end{aligned}\right. \end{aligned}$$

Through the layer-by-layer factorization process shown in Eq. (2), the original sample matrix ***X*** is decomposed into the product of *l*+1 matrices, as demonstrated in Eq. (3).

3 $$\begin{aligned} \begin{aligned} {{\varvec{X}}}={{\varvec{W}}}_l {{\varvec{H}}}_l {{\varvec{H}}}_{l-1} \ldots {{\varvec{H}}}_2 {{\varvec{H}}}_1 \end{aligned} \end{aligned}$$

where ${{\varvec{X}}} \in \mathbb {R}_{+}^{m \times n}$ represents the training sample matrix, ${{\varvec{W}}}_i \in \mathbb {R}_{+}^{m \times l_{i}}$ denotes the basis matrix at the *i*-th layer, and ${{\varvec{H}}}_i \in \mathbb {R}_{+}^{l_{i} \times n}$ is the corresponding coefficient matrix at the *i*-th layer. $l_{i}$ denotes the dimension of the *i*-th level matrix decomposition. According to the layer-wise decomposition scheme, the basis matrix ${{\varvec{W}}}_i$ is progressively factorized until the bottom-level basis ${{\varvec{W}}}_l$, which captures the deep structural features, is obtained. Ultimately, the original sample matrix ${{\varvec{X}}}$ is factorized into the basis matrix ${{\varvec{W}}}_l$ and a series of coefficient matrices $\{{{\varvec{H}}}_1, {{\varvec{H}}}_2, \dots , {{\varvec{H}}}_l\}$. In this process, all elements of the basis matrix and the coefficient matrices are non-negative. Researchers achieve the representation of the *i*-th layer basis and coefficient matrices for ${{\varvec{X}}}$ by optimizing the following objective function:

4 $$\begin{aligned} &\min _{{{\varvec{W}}}_i, {{\varvec{H}}}_i}\left\| {{\varvec{X}}}-{{\varvec{W}}}_l {{\varvec{H}}}_l \cdots {{\varvec{H}}}_2 {{\varvec{H}}}_1\right\| _\textrm{F}^2 \quad \\&\text{ s.t. } \quad {{\varvec{W}}}_i \,\,\text{and}\,\, {{\varvec{H}}}_i\,\,\text{are non-negative} \end{aligned} $$

BDNMF iteratively optimizes the factorized basis matrices to extract deep local features from the original samples. This model effectively captures the underlying local features within the data space, enabling more accurate sample representations. It has proven effective in applications like facial recognition, demonstrating its capability in extracting essential features from complex datasets.

**Algorithm 1:**
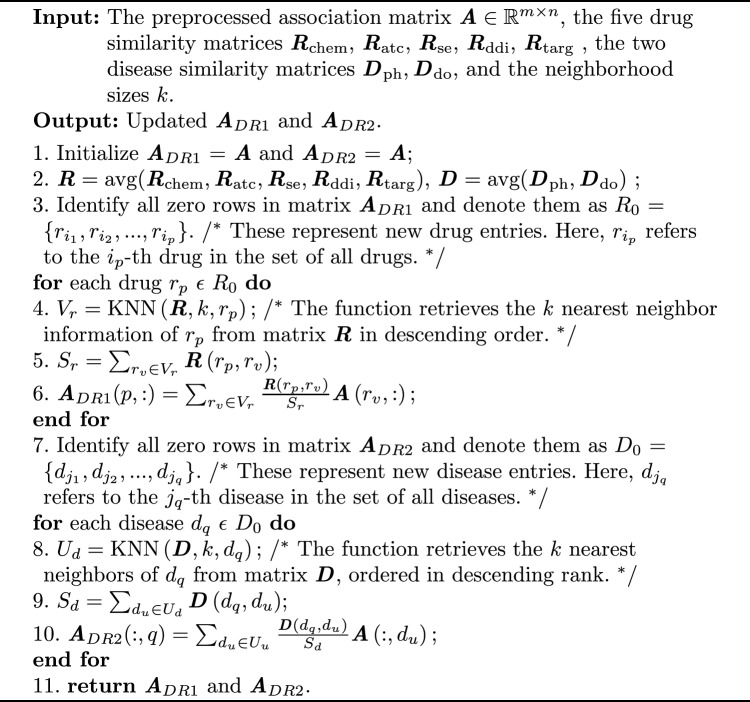
KNN Preprocessing Algorithm

### DNMF-DDA for Drug Repositioning

In constructing the drug integration matrix, we specifically focused on new drug nodes that have not established connections with any known drugs (e.g., $r_2$ in Fig. 1b, thereby enhancing the model’s applicability. These new nodes result in corresponding rows of the association matrix becoming zero vectors, thereby increasing the difficulty of matrix completion and affecting the accuracy of predictions. Inspired by [42], we employed KNN preprocessing to address this cold start problem. The specific steps are as follows: for each new drug node $r_p$, we select its *k*-nearest disease nodes and arrange them in descending order based on similarity. Subsequently, the row vector for drug $r_p$ in the association matrix is updated by incorporating weighted association information, with detailed steps outlined in Algorithm 1. After KNN preprocessing, we obtained the updated DDA matrix ${{\varvec{A}}}_{RD1}$. Similarly, we applied KNN preprocessing to new disease nodes to create a new association matrix ${{\varvec{A}}}_{RD2}$. Finally, we constructed the integration matrix for drugs and diseases by combining the comprehensive similarity matrix with the updated association matrix.

After preprocessing the association matrix, we further integrated the composite similarity matrix and the association matrix to obtain two integrated matrices for drugs and diseases (i.e., ${{\varvec{X}}}_R=[{{\varvec{R}}}, {{\varvec{A}}}_{RD1}] \in \mathbb {R}^{m \times (m+n) }$ and ${{\varvec{X}}}_D=[{{\varvec{D}}}, {{\varvec{A}}}^{\textrm{T}}_{RD2}] \in \mathbb {R}^{n \times (m+n) }$). To fully exploit the low-rank structure of these integrated matrices for deep feature extraction and matrix completion, we developed the DNMF-DDA model to predict potential drug indications. The model is as follows:

5 $$\begin{aligned} \begin{aligned}&\min _{{{\varvec{H}}}_{i}, {~{{\varvec{W}}}_{i}}} \left\| {{\varvec{X}}}-{{\varvec{W}}}_l {{\varvec{H}}}_l {{\varvec{H}}}_{l-1} \cdots {{\varvec{H}}}_1\right\| _\textrm{F}^2\\&+\alpha \sum _{i=1}^l \operatorname {Tr}\left( {{\varvec{W}}}_i^\textrm{T} {{\varvec{L}}} {{\varvec{W}}}_i\right) \\&+\beta \sum _{i=1}^{l-1}\left\| {{\varvec{W}}}_i-{{\varvec{W}}}_{i+1} {{\varvec{H}}}_{i+1}\right\| _\textrm{F}^2 \\&+\lambda \sum _{i=1}^l\left\| {{\varvec{W}}}_{i}\right\| _\textrm{F}^2+\mu \sum _{i=1}^l\left\| {{\varvec{H}}}_{i}\right\| _\textrm{F}^2 \\&\text{ s.t. } {{\varvec{W}}}_{i}\,\, \text{and}\,\, {{\varvec{H}}}_{i} \,\, \text{are negative} \end{aligned} \end{aligned}$$

Where ${{\varvec{X}}}$ is the integrated matrix obtained by consolidating similarity information and the association matrix (i.e., ${{\varvec{X}}}_R$ or ${{\varvec{X}}}_D$). To capture the local features among nodes, we introduce the graph Laplacian of the comprehensive similarity to preserve the manifold structure of the data. For instance, from the perspective of drugs, the Laplacian matrix of the comprehensive similarity matrix ${{\varvec{R}}}$ is represented as ${{\varvec{L}}} = {{\varvec{U}}} - {{\varvec{R}}}$. Here, ${{\varvec{U}}}$ is a diagonal matrix where the *j*-th diagonal entry is defined as $\sum _{i=1}^m {{\varvec{R}}}_{ji}$. The same approach can be applied to the integrated matrix from the disease perspective to capture the relationships among disease nodes. Additionally, we incorporate corresponding relaxed regularization terms for each layer’s factorization process to ensure the stability of the model. The model combines Laplacian-based local constraints with each layer’s error terms to optimize local features step by step, gradually uncovering complex relational attributes within the graph structure. This joint optimization strategy enhances the model’s capacity to extract deep local graph features.

### Solutions for DNMF-DDA

To facilitate factor matrix approximation, we pre-train each layer with the BasicNMF algorithm, yielding initial estimates. The pre-training begins by decomposing the adjacency matrix as ${{\varvec{X}}} \approx {{\varvec{W}}}_1 {{\varvec{H}}}_1$, where ${{\varvec{W}}}_1 \in \mathbb {R}^{m \times l _1}_+$ and ${{\varvec{H}}}_1 \in \mathbb {R}^{l _1 \times n}_+$. Subsequently, ${{\varvec{W}}}_1$ is further factorized into ${{\varvec{W}}}_1 \approx {{\varvec{W}}}_2 {{\varvec{H}}}_2$, with ${{\varvec{W}}}_2 \in \mathbb {R}^{m \times l _2}_+$ and ${{\varvec{H}}}_2 \in \mathbb {R}^{l _2 \times l _1}_+$. This step is repeated for each layer until all layers are pre-trained. The matrix awaiting the first decomposition is denoted as ${{\varvec{X}}}_R$ or ${{\varvec{X}}}_D$, and the matrix awaiting the $i$-th decomposition is denoted as ${{\varvec{W}}}_{i-1}$. Once pre-training is complete, we update ${{\varvec{W}}}_i$ and ${{\varvec{H}}}_i$ in the objective function using the Lagrangian multiplier model. To satisfy the non-negativity constraints of ${{\varvec{W}}}_i$ and ${{\varvec{H}}}_i$, we incorporate two Lagrangian multipliers, $\varTheta$ and $\varOmega$, resulting in the following objective function:

6 $$\begin{aligned} \begin{aligned} \min _{{{\varvec{W}}}_i, {{\varvec{H}}}_i} \mathcal {L}\left( {{\varvec{W}}}_i, {{\varvec{H}}}_i\right)&=\left\| {{\varvec{X}}}-{{\varvec{W}}}_l {{\varvec{H}}}_l {{\varvec{H}}}_{l-1} \cdots {{\varvec{H}}}_1\right\| _\text{F}^2 \\&+\alpha \sum _{i=1}^l \operatorname {Tr}\left( {{\varvec{W}}}_i^\text{T} {{\varvec{L}}} {{\varvec{W}}}_i\right) \\&+\beta \sum _{i=1}^{l-1}\left\| {{\varvec{W}}}_i-{{\varvec{W}}}_{i+1} {{\varvec{H}}}_{i+1}\right\| _\text{F}^2 \\&+\lambda \sum _{i=1}^l\left\| {{\varvec{W}}}_{i}\right\| _\text{F}^2+\mu \sum _{i=1}^l\left\| {{\varvec{H}}}_{i}\right\| _\text{F}^2\\&+\operatorname {Tr}\left(\it{ \varTheta }{{\varvec{W}}}_i^\text{T}\right) +\operatorname {Tr}\left( \it{\varOmega} {{\varvec{H}}}_i^\text{T}\right) \end{aligned} \end{aligned}$$

**Updating** ${{\varvec{W}}}_i$ ($i=1$): **to update** ${{\varvec{W}}}_i$ **with** ${{\varvec{H}}}_i$ **fix.** Equation (6) is rewritten as follows:

7 $$\begin{aligned} \begin{aligned} \mathcal {L}\left( {{\varvec{W}}}_i\right)&=\left\| {{\varvec{X}}}-{{\varvec{W}}}_i {\varvec{\varPhi }}_i\right\| _\textrm{F}^2+\alpha \operatorname {Tr}\left( {{\varvec{W}}}_i^\textrm{T} {{\varvec{L}}} {{\varvec{W}}}_i\right) \\&+\beta \left\| {{\varvec{W}}}_i-{{\varvec{W}}}_{i+1} {{\varvec{H}}}_{i+1}\right\| _\textrm{F}^2 +\lambda \left\| {{\varvec{W}}}_i\right\| _\textrm{F}^2+\operatorname {Tr}\left( \it{\varTheta} {{\varvec{W}}}_i^\textrm{T}\right) \end{aligned} \end{aligned}$$

where ${\varvec{\varPhi }}_i=\prod _{p=1}^i {{\varvec{H}}}_{i-p+1}$. When $i = 1$, set ${\varvec{\varPhi }}_i = {{\varvec{H}}}_1$. Then, rewrite Eq. (7) as follows:

8 $$\begin{aligned} \begin{aligned} \mathcal {L}\left( {{\varvec{W}}}_i,\it{ \varTheta} \right)&= -2\operatorname {Tr}\left( {{\varvec{X}}}^\textrm{T} {{\varvec{W}}}_i {\varvec{\varPhi }}_i \right) + \operatorname {Tr}\left( {\varvec{\varPhi }}^\textrm{T}_i {{\varvec{W}}}_i^\textrm{T} {{\varvec{W}}}_i {\varvec{\varPhi }}_i \right) \\&+\alpha \operatorname {Tr}\left( {{\varvec{W}}}_i^\textrm{T} {{\varvec{L}}} {{\varvec{W}}}_i\right) \\&+\beta \operatorname {Tr}\left( {{\varvec{W}}}_i^\textrm{T} {{\varvec{W}}}_i\right) - 2 \beta \operatorname {Tr}\left( {{\varvec{W}}}_i^\textrm{T} {{\varvec{W}}}_{i+1} {{\varvec{H}}}_{i+1}\right) \\&+\lambda \operatorname {Tr}\left( {{\varvec{W}}}_i^\textrm{T} {{\varvec{W}}}_i\right) +\operatorname {Tr}\left( \varTheta {{\varvec{W}}}_i^\textrm{T}\right) \end{aligned} \end{aligned}$$

To solve this, we set the partial derivative $\frac{\partial \mathcal {L}\left( {{\varvec{W}}}_i, \it{\varTheta} \right) }{\partial {{\varvec{W}}}_i} = 0$ and apply the Karush-Kuhn-Tucker (KKT) condition $\it{\varTheta} _{k l} ({{\varvec{W}}}_i)_{k l} = 0$. We obtain the following expression:

9 $$\begin{aligned} \begin{aligned}&\left( -{{\varvec{X}}} {\varvec{\varPhi }}_i^\textrm{T} + {{\varvec{W}}}_i {\varvec{\varPhi }}_i {\varvec{\varPhi }}_i^\textrm{T}+\alpha {{\varvec{L}}} {{\varvec{W}}}_i \right. \\&\left. +(\beta +\lambda ) {{\varvec{W}}}_i-\beta {{\varvec{W}}}_{i+1} {{\varvec{H}}}_{i+1}\right) _{k l}\left( {{\varvec{W}}}_i\right) _{k l}^2=0 \end{aligned} \end{aligned}$$

Equation (9) can be solved using the following update rule:

10 $$\begin{aligned} \begin{aligned} {{\varvec{W}}}_i={{\varvec{W}}}_i \sqrt{\frac{\left[ {{\varvec{X}}} {\varvec{\varPhi }}_i^\textrm{T}\right] ^{+}+\left[ {{\varvec{W}}}_i {\varvec{\varPhi }}_i {\varvec{\varPhi }}_i^\textrm{T}\right] ^{-}+\alpha \left[ {{\varvec{L}}} {{\varvec{W}}}_i\right] ^{-}+(\beta +\lambda )\left[ {{\varvec{W}}}_i\right] ^{-}+\beta \left[ {{\varvec{W}}}_{i+1} {{\varvec{H}}}_{i+1}\right] ^{+}}{\left[ {{\varvec{X}}} {\varvec{\varPhi }}_i^\textrm{T}\right] ^{-}+\left[ {{\varvec{W}}}_i {\varvec{\varPhi }}_i {\varvec{\varPhi }}_i^\textrm{T}\right] ^{+}+\alpha \left[ {{\varvec{L}}} {{\varvec{W}}}_i\right] ^{+}+(\beta +\lambda )\left[ {{\varvec{W}}}_i\right] ^{+}+\beta \left[ {{\varvec{W}}}_{i+1} {{\varvec{H}}}_{i+1}\right] ^{-}}} \end{aligned} \end{aligned}$$

where $[{{\varvec{M}}}]^+$ represents the matrix with all negative elements replaced by 0, while $[{{\varvec{M}}}]^-$ corresponds to the matrix with all positive elements set to 0. Their definitions are as follows:

11 $$\begin{aligned} ~ [{{\varvec{M}}}]_{i, j}^{+}=\frac{\left| {{\varvec{M}}}_{i j}\right| +{{\varvec{M}}}_{i j}}{2},\,\,[{{\varvec{M}}}]_{i, j}^{-}=\frac{\left| {{\varvec{M}}}_{i j}\right| -{{\varvec{M}}}_{i j}}{2},\,\forall i, j  \end{aligned}$$

**Updating** ${{\varvec{W}}}_i$ ($1< i < l$): ** to update** ${{\varvec{W}}}_i$ **with** ${{\varvec{H}}}_i$ **fix.** The Lagrangian function is defined as follows:

12 $$\begin{aligned} \begin{aligned} \mathcal {L}\left( {{\varvec{W}}}_i,\it{ \varTheta }\right)&= -2\operatorname {Tr}\left( {{\varvec{X}}}^\textrm{T} {{\varvec{W}}}_i {\varvec{\varPhi }}_i \right) + \operatorname {Tr}\left( {\varvec{\varPhi }}^\textrm{T}_i {{\varvec{W}}}_i^\textrm{T} {{\varvec{W}}}_i {\varvec{\varPhi }}_i \right) \\ &+\alpha \operatorname {Tr}\left( {{\varvec{W}}}_i^\textrm{T} {{\varvec{L}}} {{\varvec{W}}}_i\right) \\&-2 \beta \operatorname {Tr}\left( {{\varvec{W}}}_{i-1}^\textrm{T} {{\varvec{W}}}_{i} {{\varvec{H}}}_{i}\right) \\ &+\beta \operatorname {Tr}\left( {{\varvec{H}}}_i^\textrm{T} {{\varvec{W}}}_i^\textrm{T} {{\varvec{W}}}_i {{\varvec{H}}}_i\right) +\beta \operatorname {Tr}\left( {{\varvec{W}}}_i^\textrm{T} {{\varvec{W}}}_i\right) \\&-2 \beta \operatorname {Tr}\left( {{\varvec{W}}}_i^\textrm{T} {{\varvec{W}}}_{i+1} {{\varvec{H}}}_{i+1}\right) +\lambda \operatorname {Tr}\left( {{\varvec{W}}}_i^\textrm{T} {{\varvec{W}}}_i\right) \\&+\operatorname {Tr}\left( \it{\varTheta} {{\varvec{W}}}_i^\textrm{T}\right) \end{aligned} \end{aligned}$$

Using the same computational approach, we can derive the following results:

13 $$\begin{aligned} \begin{aligned} {{\varvec{W}}}_i={{\varvec{W}}}_i \sqrt{\frac{\left[ {{\varvec{X}}} {\varvec{\varPhi }}_i^\textrm{T}\right] ^{+}+\left[ {{\varvec{W}}}_i {\varvec{\varPhi }}_i {\varvec{\varPhi }}_i^\textrm{T}\right] ^{-}+\alpha \left[ {{\varvec{L}}} {{\varvec{W}}}_i\right] ^{-}+(\beta +\lambda )\left[ {{\varvec{W}}}_i\right] ^{-}+\beta \left[ {{\varvec{W}}}_{i-1} {{\varvec{H}}}^\textrm{T}_{i}\right] ^{+}+\beta \left[ {{\varvec{W}}}_{i} {{\varvec{H}}}_{i} {{\varvec{H}}}^\textrm{T}_{i}\right] ^{-}+\beta \left[ {{\varvec{W}}}_{i+1} {{\varvec{H}}}_{i+1}\right] ^{+}}{\left[ {{\varvec{X}}} {\varvec{\varPhi }}_i^\textrm{T}\right] ^{-}+\left[ {{\varvec{W}}}_i {\varvec{\varPhi }}_i {\varvec{\varPhi }}_i^\textrm{T}\right] ^{+}+\alpha \left[ {{\varvec{L}}} {{\varvec{W}}}_i\right] ^{+}+(\beta +\lambda )\left[ {{\varvec{W}}}_i\right] ^{+}+\beta \left[ {{\varvec{W}}}_{i-1} {{\varvec{H}}}^\textrm{T}_{i}\right] ^{-}+\beta \left[ {{\varvec{W}}}_{i} {{\varvec{H}}}_{i} {{\varvec{H}}}^\textrm{T}_{i}\right] ^{+}+\beta \left[ {{\varvec{W}}}_{i+1} {{\varvec{H}}}_{i+1}\right] ^{-}}} \end{aligned} \end{aligned}$$

**Updating** ${{\varvec{W}}}_i$ ($i = l$): **to update** ${{\varvec{W}}}_i$ **with** ${{\varvec{H}}}_i$ **fix.** The Lagrangian function is expressed as

14 $$\begin{aligned} \begin{aligned} \mathcal {L}\left( {{\varvec{W}}}_i, \it{\varTheta} \right)&= -2\operatorname {Tr}\left( {{\varvec{X}}}^\textrm{T} {{\varvec{W}}}_i {\varvec{\varPhi }}_i \right) + \operatorname {Tr}\left( {\varvec{\varPhi }}^\textrm{T}_i {{\varvec{W}}}_i^\textrm{T} {{\varvec{W}}}_i {\varvec{\varPhi }}_i \right) \\&+\alpha \operatorname {Tr}\left( {{\varvec{W}}}_i^\textrm{T} {{\varvec{L}}} {{\varvec{W}}}_i\right) \\&+\beta \operatorname {Tr}\left( {{\varvec{H}}}_i^\textrm{T} {{\varvec{W}}}_i^\textrm{T} {{\varvec{W}}}_i {{\varvec{H}}}_i\right) - 2 \beta \operatorname {Tr}\left( {{\varvec{W}}}^\textrm{T}_{i-1} {{\varvec{W}}}_i {{\varvec{H}}}_i\right) \\ &+\lambda \operatorname {Tr}\left( {{\varvec{W}}}_i^\textrm{T} {{\varvec{W}}}_i\right) \\&+\operatorname {Tr}\left( \it{\varTheta} {{\varvec{W}}}_i^\textrm{T}\right) \end{aligned} \end{aligned}$$

Similarly, the following result is obtained:

15 $$\begin{aligned} \begin{aligned} {{\varvec{W}}}_i={{\varvec{W}}}_i \sqrt{\frac{\left[ {{\varvec{X}}} {\varvec{\varPhi }}_i^\textrm{T}\right] ^{+}+\left[ {{\varvec{W}}}_i {\varvec{\varPhi }}_i {\varvec{\varPhi }}_i^\textrm{T}\right] ^{-}+\alpha \left[ {{\varvec{L}}} {{\varvec{W}}}_i\right] ^{-}+\lambda \left[ {{\varvec{W}}}_i\right] ^{-}+\beta \left[ {{\varvec{W}}}_{i-1} {{\varvec{H}}}^\textrm{T}_{i}\right] ^{+}+\beta \left[ {{\varvec{W}}}_{i} {{\varvec{H}}}_{i} {{\varvec{H}}}^\textrm{T}_{i}\right] ^{-}}{\left[ {{\varvec{X}}} {\varvec{\varPhi }}_i^\textrm{T}\right] ^{-}+\left[ {{\varvec{W}}}_i {\varvec{\varPhi }}_i {\varvec{\varPhi }}_i^\textrm{T}\right] ^{+}+\alpha \left[ {{\varvec{L}}} {{\varvec{W}}}_i\right] ^{+}+\lambda \left[ {{\varvec{W}}}_i\right] ^{+}+\beta \left[ {{\varvec{W}}}_{i-1} {{\varvec{H}}}^\textrm{T}_{i}\right] ^{-}+\beta \left[ {{\varvec{W}}}_{i} {{\varvec{H}}}_{i} {{\varvec{H}}}^\textrm{T}_{i}\right] ^{+}}} \end{aligned} \end{aligned}$$

**Updating** ${{\varvec{H}}}_i$ ($i = 1$): **to update** ${{\varvec{H}}}_i$ **with** ${{\varvec{W}}}_i$ **fix.** Equation (6) is rewritten as follows:

16 $$\begin{aligned} \begin{aligned} \mathcal {L}\left( {{\varvec{H}}}_i, \varOmega \right)&= - 2\operatorname {Tr}\left( {{\varvec{X}}}^\textrm{T} {{\varvec{W}}}_i {{\varvec{H}}}_i \right) + \operatorname {Tr}\left( {{\varvec{H}}}^\textrm{T}_i {{\varvec{W}}}^\textrm{T}_i {{\varvec{W}}}_i {{\varvec{H}}}_i \right) \\&+\mu \operatorname {Tr}\left( {{\varvec{H}}}^\textrm{T}_i {{\varvec{H}}}_i \right) +\operatorname {Tr}\left( \varOmega {{\varvec{H}}}_i^\textrm{T}\right) . \end{aligned} \end{aligned}$$

We set $\frac{\partial \mathcal {L}\left( {{\varvec{H}}}_i, \it{\varOmega} \right) }{\partial {{\varvec{H}}}_i} = 0$ and utilize the KKT conditions to solve Eq. (16):

17 $$\begin{aligned} \begin{aligned} {{\varvec{H}}}_i={{\varvec{H}}}_i \sqrt{\frac{\left[ {{\varvec{W}}}^\textrm{T}_i {{\varvec{W}}}_i {{\varvec{H}}}_i\right] ^{-}+\left[ {{\varvec{W}}}^\textrm{T}_i {{\varvec{X}}}\right] ^{+}+\mu \left[ {{\varvec{H}}}_i\right] ^{-}}{\left[ {{\varvec{W}}}^\textrm{T}_i {{\varvec{W}}}_i {{\varvec{H}}}_i\right] ^{+}+\left[ {{\varvec{W}}}^\textrm{T}_i {{\varvec{X}}}\right] ^{-}+\mu \left[ {{\varvec{H}}}_i\right] ^{+}}} \end{aligned} \end{aligned}$$

**Updating** ${{\varvec{H}}}_i$ ($1 < i \le l$): **to update** ${{\varvec{H}}}_i$ **with** ${{\varvec{W}}}_i$ **fix.** The Lagrangian function is defined as follows:

18 $$\begin{aligned} \begin{aligned} \mathcal {L}\left( {{\varvec{H}}}_i, \it{\varOmega} \right)&= - 2\operatorname {Tr}\left( {{\varvec{X}}}^\textrm{T} {{\varvec{W}}}_i {{\varvec{H}}}_i {\varvec{\varPhi }}_{i-1} \right) \\&+ \operatorname {Tr}\left( {\varvec{\varPhi }}^\textrm{T}_{i-1} {{\varvec{H}}}^\textrm{T}_i {{\varvec{W}}}^\textrm{T}_i {{\varvec{W}}}_i {{\varvec{H}}}_i {\varvec{\varPhi }}_{i-1} \right) \\&- 2\beta \operatorname {Tr}\left( {{\varvec{W}}}^\textrm{T}_{i-1} {{\varvec{W}}}_i {{\varvec{H}}}_i \right) \\ &+ \beta \operatorname {Tr}\left( {{\varvec{H}}}^\textrm{T}_i {{\varvec{W}}}^\textrm{T}_i {{\varvec{W}}}_i {{\varvec{H}}}_i\right) +\mu \operatorname {Tr}\left( {{\varvec{H}}}^\textrm{T}_i {{\varvec{H}}}_i \right) \\&+\operatorname {Tr}\left( \it{\varOmega} {{\varvec{H}}}_i^\textrm{T}\right) \end{aligned} \end{aligned}$$

We can obtain the following results using the same computational method:

19 $$\begin{aligned} \begin{aligned} {{\varvec{H}}}_i={{\varvec{H}}}_i \sqrt{\frac{\left[ {{\varvec{W}}}^\textrm{T}_i {{\varvec{W}}}_i {{\varvec{H}}}_i {\varvec{\varPhi }}_{i-1} {\varvec{\varPhi }}^\textrm{T}_{i-1}\right] ^{-}+\left[ {{\varvec{W}}}^\textrm{T}_i {{\varvec{X}}} {\varvec{\varPhi }}^\textrm{T}_{i-1} \right] ^{+}+\mu \left[ {{\varvec{H}}}_i\right] ^{-}+ \beta \left[ {{\varvec{W}}}^\textrm{T}_i {{\varvec{W}}}_{i-1} \right] ^{+}+ \beta \left[ {{\varvec{W}}}^\textrm{T}_i {{\varvec{W}}}_i {{\varvec{H}}}_i \right] ^{-}}{\left[ {{\varvec{W}}}^\textrm{T}_i {{\varvec{W}}}_i {{\varvec{H}}}_i {\varvec{\varPhi }}_{i-1} {\varvec{\varPhi }}^\textrm{T}_{i-1}\right] ^{+}+\left[ {{\varvec{W}}}^\textrm{T}_i {{\varvec{X}}} 
{\varvec{\varPhi }}^\textrm{T}_{i-1}\right] ^{-}+\mu \left[ {{\varvec{H}}}_i\right] ^{+}+ \beta \left[ {{\varvec{W}}}^\textrm{T}_i {{\varvec{W}}}_{i-1} \right] ^{-}+ \beta \left[ {{\varvec{W}}}^\textrm{T}_i {{\varvec{W}}}_i {{\varvec{H}}}_i \right] ^{+}}} \end{aligned} \end{aligned}$$

By iteratively updating ${{\varvec{W}}}_i$ and ${{\varvec{H}}}_i$ according to the formulas above until convergence, the DNMF-DDA model can be optimized. As an example, we take the integrated matrix from the drug perspective. Through this optimization, DNMF-DDA can extract the deep low-rank features of drugs. The refined integrated matrix ${{\varvec{X}}}_R^* \in \mathbb {R}_{+}^{m \times (m+n)}$ is obtained by back-filling based on the optimized basis and coefficient matrices.

20 $$\begin{aligned} \begin{aligned} {{\varvec{X}}}^*_R={{\varvec{W}}}_{R_{l}}{{\varvec{H}}}_{R_{l}}{{\varvec{H}}}_{R_{l-1}}\cdots {{\varvec{H}}}_{R_{1}} \end{aligned} \end{aligned}$$

where, ${{\varvec{W}}}_{R_{i}}$ and ${{\varvec{H}}}_{R_{i}}$ represent the basis matrix and the coefficient matrix, respectively, obtained from the $i$-th layer decomposition of ${{\varvec{X}}}_R$. The focus is on the association information in ${{\varvec{X}}}_R^*$, represented as ${{\varvec{A}}}_{RD1}^* = {{\varvec{X}}}_R^*(:, m+1:\textrm{end})$. Similarly, an optimized integrated matrix ${{\varvec{X}}}_D^* \in \mathbb {R}_{+}^{n \times (m+n) }$ can be obtained from the disease perspective as well, and its association information is denoted as ${{\varvec{A}}}_{RD2}^* = {[{{\varvec{X}}}_D^*(:, n+1:\textrm{end})]}^\textrm{T}$. The final association matrix ${{\varvec{A}}}_{RD}^*$ is computed by averaging the predictions from both drug and disease perspectives as ${{\varvec{A}}}_{RD}^* = \text {avg}({{\varvec{A}}}_{RD1}^*$, ${{\varvec{A}}}_{RD2}^*)$. Algorithm 2 outlines the procedure for applying DNMF-DDA. The repository containing the code and data can be accessed at https://github.com/YangPhD84/DNMFDDA.

**Algorithm 2:**
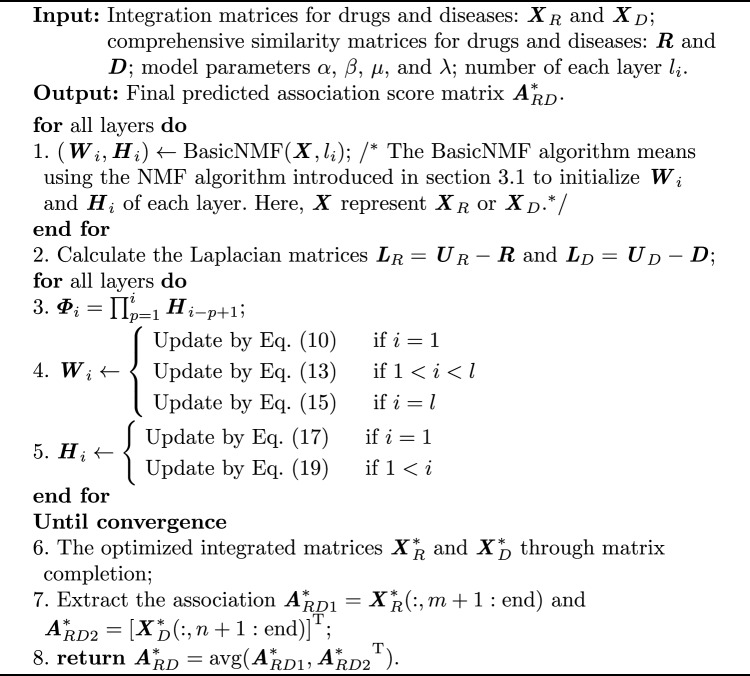
Solution for DNMF-DDA Model.

## Results

### Evaluation Metrics

We adopted cold start testing and 10-fold cross-validation (10-fold CV) as experimental strategies to rigorously evaluate the DNMF-DDA model’s capacity to predict novel drugs and their indications across diverse datasets. In the cold-start tests, drugs with only one known disease association were considered target drugs. To evaluate the algorithm’s performance under cold-start conditions, each target drug is sequentially used as the test subject, while all other known associations serve as the training set. By repeating this process, comprehensive performance metrics are ultimately obtained. To simulate testing on new drugs, similarity data that have not yet been tested for these drugs (i.e., ${{\varvec{R}}}_{\textrm{ddi}}$ and ${{\varvec{R}}}_{\textrm{se}}$) were excluded. For the cold-start experiment, only three drug similarity metrics and two disease similarity metrics were used. During 10-fold CV, the dataset is partitioned into ten subsets, with each subset alternately serving as the training set while the remaining nine are used for validation. This approach minimizes data partitioning bias, enhancing the stability of model evaluation and ensuring robust performance across diverse datasets. To quantify the performance of the DNMF-DDA model, we employed three evaluation metrics: AUC (area under the receiver operating characteristic curve), AUPR (area under the precision-recall curve), and precision, which assess the model’s overall predictive ability, its performance under conditions of data imbalance, and prediction correctness, respectively. Through these two experimental designs and a comprehensive analysis of multiple evaluation metrics, we effectively assessed the performance of the DNMF-DDA model, providing a quantitative basis for its optimization and practical application.

### Parameters Setting

In this study, key parameters to be defined include the *k*-neighborhood value, $\alpha$, $\beta$, $\mu$, $\lambda$, and the size of the deep layers. Since the framework utilizes a multi-layer deep structure for link prediction, it is necessary to define the number of layers for all datasets. We define the dimensions of ${{\varvec{W}}}_i$ and ${{\varvec{H}}}_i$ as $l ^{~(\tau _i)}_i = [\tau _i \min (m,n)]$, where $l ^{~(\tau _i)}_i$ represents the dimensionality of the *i*-th layer, $\tau _i \in [0, 1]$, and $[\cdot ]$ denotes the rounding function. For instance, in this setup, $l _2^{~(0.6)}$ signifies that the second layer’s decomposition dimension is 0.6 times the original matrix size. This configuration allows flexibility in matching the final latent space based on the characteristics of the original data. To mitigate overfitting caused by excessive parameters, we set $\mu$ and $\lambda$ to the same value.

**Fig. 3:**
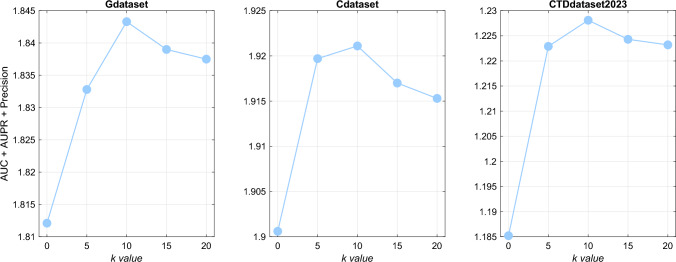
Sensitivity analysis plots of *k* values across different datasets

When optimizing these key parameters, we fixed other parameters to investigate the effect of a specific parameter on the model. For instance, we set $k = 10$, $\alpha = 0.01$, $\beta = 1$, and $\mu = 1$, and configured the model to have 2 layers to examine the factors influencing the final latent space. Through cross-validation, we selected $\alpha$, $\beta$, and $\mu$ from $\{0.001, 0.01, 0.1, 1, 10\}$ and $\tau _i$ from $\{0.2, 0.3, 0.4, 0.5, 0.6, 0.7, 0.8\}$. On this basis, we fixed other parameters and selected *k* values from $\{5, 10, 15, 20\}$ to evaluate DNMF-DDA’s performance across different *k* values. As shown in Fig. 3, a comprehensive analysis of parameter *k* was conducted across all datasets. The results indicate that the weighted KNN preprocessing enhances the DNMF-DDA model’s ability to extract deep key features, achieving optimal performance around $k = 10$. Although excessively large *k* values introduce noise, the overall performance remains stable, demonstrating the model’s robustness to parameter variations. Our exploration and calculations indicated that the optimal performance was achieved in Gdataset and Cdataset with $k = 10$, $\alpha = 0.01$, $\beta = \mu = \lambda = 1$, and layer sizes of $[l _1^{~(0.8)}, l _2^{~(0.6)}]$. In the CTDdataset2023, optimal performance was similarly achieved with $k = 10$, $\alpha = \beta = \mu = \lambda = 1$, and the same layer sizes.

Simultaneously, the DNMF-DDA model iteration terminates upon satisfying the stopping criteria or reaching the maximum iteration limit:

21 $$\begin{aligned} h_{k}\le \xi _1, \frac{|h_{k+1}-h_{k}|}{ max\{1,|h_{k}|\} } \le \xi _2 \end{aligned}$$

where $h_{k} = \frac{{ \left\| ({{\varvec{A}}}^*_{DR})_{k+1} - ({{\varvec{A}}}^*_{DR})_{k}\right\| }_{\textrm{F}}}{{\left\| ({{\varvec{A}}}^*_{DR})_{k}\right\| }_{\textrm{F}}}$, and $\xi _1$ and $\xi _2$ represent the specified tolerances. In this context, $\xi _1$ is set to $5 \times 10^{-4}$ and $\xi _2$ is established at $5 \times 10^{-7}$.

### Compared to Multiple Methods of Drug Repositioning

This study evaluates DNMF-DDA against five cutting-edge drug repositioning methods: DDA-SKF [43], MSBMF [24], HGIMC [44], BNNR [23], and MBiRW [10]. Each of these baseline methods is configured with its optimal parameters. To assess the predictive capabilities of these computational models and their potential for identifying novel drug indications, we conduct both the cold-start test and the 10-fold CV across three datasets (i.e., Gdataset, Cdataset, and CTDdataset2023). The best and second-best results are displayed in bold and underlined, respectively.

**Table 2:** The cold-start test results for all models in each dataset

Datasets	Metrics	**DNMF-DDA**	DDA-SKF	MSBMF	HGIMC	MBiRW	BNNR
Gdataset	AUC	${\textbf {0.921}}$	0.832	0.875	$\underline{0.877}$	0.818	0.830
	AUPR	${\textbf {0.361}}$	0.256	0.301	$\underline{0.356}$	0.189	0.199
	Precision	$\underline{0.421}$	0.310	0.368	${\textbf {0.433}}$	0.234	0.251
Cdataset	AUC	${\textbf {0.921}}$	0.832	$\underline{0.883}$	0.879	0.804	0.812
	AUPR	$\underline{0.295}$	0.200	${\textbf {0.298}}$	0.294	0.174	0.193
	Precision	$\underline{0.362}$	0.246	${\textbf {0.367}}$	0.356	0.232	0.254
CTDdataset2023	AUC	${\textbf {0.918}}$	0.879	$\underline{0.904}$	0.890	0.883	0.816
	AUPR	${\textbf {0.240}}$	$\underline{0.233}$	0.190	0.222	0.118	0.111
	Precision	${\textbf {0.283}}$	$\underline{0.277}$	0.219	0.267	0.139	0.137

Optimal values are in bold and the suboptimal values are in underline

The cold-start test results are presented in Table 2. DNMF-DDA consistently surpasses all baseline methods on all datasets in AUC, demonstrating significant and sustained improvements. Specifically, the AUC scores for DNMF-DDA exceed 0.915 in all three datasets, outperforming the second-best model by $5.26\%$, $4.30\%$, and $1.22\%$ on Gdataset, Cdataset, and CTDdataset2023, respectively. In terms of AUPR, our model also delivers strong results, achieving the highest performance on Gdataset and CTDdataset2023, and securing the second-best performance on Cdataset, just behind MSBMF. Additionally, DNMF-DDA demonstrates excellent precision across all datasets. These results indicate that the DNMF-DDA model demonstrates superior robustness and generalization in cold-start scenarios. DNMF-DDA leverages the deep non-negative matrix factorization to integrate global similarities and local structural information while progressively optimizing features through multi-layer factorization. This optimization compensates for missing data by extracting deep key features, making it particularly effective in addressing cold-start problems. By extracting latent information from limited initial data, DNMF-DDA demonstrates significant advantages in predicting indications for novel drugs.

**Fig. 4:**
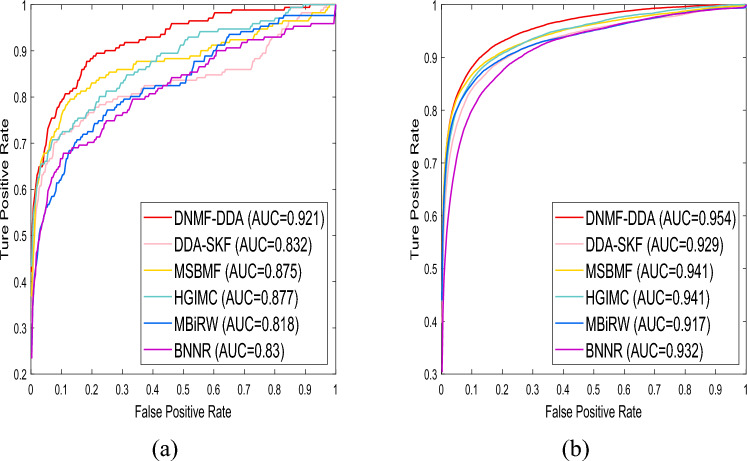
Performance evaluation of methods on Gdataset involves the 10-fold cross-validation and the cold-start test. **a** ROC curves for the cold-start test. **b** ROC curves for the 10-fold cross-validation

The outcomes of the 10-fold CV are summarized in Table 3. In terms of AUC, DNMF-DDA achieves the best performance, showing significant improvements across all datasets. Specifically, our model surpasses the second-best model by $1.38\%$, $0.94\%$, and $1.48\%$ on Gdataset, Cdataset, and CTDdataset2023, respectively. For AUPR and precision, DNMF-DDA obtains the highest scores on Gdataset and Cdataset and ranks second on CTDdataset2023, just behind DDA-SKF. The 10-fold CV results confirm that DNMF-DDA achieves the best overall performance compared to other baseline models, offering highly accurate association predictions. By progressively refining features through deep decomposition, DNMF-DDA optimizes its understanding of complex relationships within the data. This extraction process allows the model to effectively leverage latent patterns in the original data, leading to robust performance in association prediction tasks. Figures 4a and 4b respectively display ROC curves during the cold-start test and the 10-fold CV in Gdataset.

**Table 3:** The performance of all models in each dataset based on 10-fold validation

Datasets	Metrics	**DNMF-DDA**	DDA-SKF	MSBMF	HGIMC	MBiRW	BNNR
Gdataset	AUC	${\textbf {0.954}}$	0.929	$\underline{0.941}$	$\underline{0.941}$	0.917	0.932
	AUPR	${\textbf {0.424}}$	0.354	$\underline{0.421}$	0.394	0.264	0.402
	Precision	${\textbf {0.461}}$	0.397	$\underline{0.455}$	0.438	0.304	0.440
Cdataset	AUC	${\textbf {0.965}}$	0.938	$\underline{0.956}$	0.953	0.933	0.948
	AUPR	${\textbf {0.460}}$	0.392	$\underline{0.446}$	0.428	0.310	0.441
	Precision	${\textbf {0.489}}$	0.429	$\underline{0.473}$	0.463	0.351	0.471
CTDdataset2023	AUC	${\textbf {0.906}}$	0.874	$\underline{0.892}$	0.886	0.874	0.849
	AUPR	$\underline{0.148}$	${\textbf {0.168}}$	0.144	0.134	0.110	0.107
	Precision	$\underline{0.168}$	${\textbf {0.203}}$	0.164	0.155	0.128	0.121

Optimal values are in bold and the suboptimal values are in underline

However, during the 10-fold CV, DNMF-DDA exhibited lower AUPR and accuracy on CTDdataset2023 compared to the best baseline method. This discrepancy may stem from certain drugs in CTDdataset2023 that display similarity exclusively with themselves (i.e., the row sum of the similarity matrix equals 1). Such irregularities impede the effectiveness of the Laplacian regularization term in capturing local topological features. Nevertheless, despite the challenges in local feature representation, DNMF-DDA demonstrates superior overall discriminative performance, as reflected by its higher AUC.

## Deep Structure Sensitivity Experiments

Here, we investigate how the deep matrix decomposition structure impacts the model, with a focus on layer size and the number of resulting latent spaces. We perform experiments to analyze the effect of varying layer sizes on model performance. For instance, Table 4 presents the experimental results in Gdataset. As shown, when the layer sizes are set to $[l _1^{~(0.6)}]$, $[l _1^{~(0.8)}, l _2^{~(0.6)}]$, and $[l _1^{~(0.8)}, l _2^{~(0.7)}, l _3^{~(0.6)}]$, our model exhibits good stability, indicating that it is not sensitive to layer size. Conversely, the combination of $[l _1^{~(0.8)}, l _2^{~(0.6)}]$ demonstrates superior performance.

Additionally, the number of final layers may represent another sensitive parameter affecting the deep structure of DNMF-DDA. For the 2-layer model, experiments were performed by setting the final layer size as multiples of the original matrix, with $\tau _i$ selected from $\{0.2, 0.3, 0.4, 0.5, 0.6, 0.7\}$. The model’s sensitivity is analyzed through cross-validation on Gdataset, with AUC, AUPR, and precision summed to provide an overall performance measure. The results in Gdataset are shown in Table 5. The findings demonstrate that our model is highly sensitive when the number of final layers varies. Specifically, when the dimension of the final layer is too low, the model may fail to capture key information in the data, limiting its ability to extract latent features and leading to information loss that subsequently degrades model performance. In such cases, matrix decomposition may not accurately reconstruct the complexity of the data, resulting in deficiencies in identifying intrinsic patterns. Conversely, when the dimension is too high, the model may be overwhelmed by excessive noise, making it challenging to grasp the core structure of the data and potentially leading to overfitting, which negatively affects model stability and generalization. Furthermore, high-dimensional data often becomes sparse, hindering the effective utilization of the matrix’s low-rank properties and obscuring the data structure. Therefore, to balance the integrity of information with the impact of noise, we select $[l _1^{~(0.8)}, l _2^{~(0.6)}]$ as the decomposition structure for the DNMF-DDA model.

**Table 4:** Results of DNMF-DDA with different layers size in Gdataset

Tests	Metrics	Layer size		
		$[l _1^{~(0.6)}]$	$[l _1^{~(0.8)}, l_2^{~(0.6)}]$	$[l _1^{~(0.8)},l _2^{~(0.7)}, l _3^{~(0.6)}]$
10-fold CV	AUC	0.952	${\textbf {0.954}}$	0.953
	AUPR	0.421	${\textbf {0.424}}$	0.406
	Precision	0.457	${\textbf {0.461}}$	0.444
Cold-start test	AUC	0.916	${\textbf {0.921}}$	0.919
	AUPR	0.351	${\textbf {0.361}}$	0.350
	Precision	0.404	${\textbf {0.421}}$	0.404

The optimal values are in bold

**Table 5:** Results of DNMF-DDA on Gdataset under varying final layer numbers

Metrics	Layer size					
	$l _2^{~(0.7)}$	$l _2^{~(0.6)}$	$l _2^{~(0.5)}$	$l _2^{~(0.4)}$	$l _2^{~(0.3)}$	$l _2^{~(0.2)}$
AUC+AUPR+Precision	1.829	${\textbf {1.843}}$	1.823	1.816	1.795	1.772

The optimal values are in bold

**Table 6:** The comparison of the results for DNMF-DDA, DNMF-DDA (w/o KNN), DNMF-DDA (Single), and DNMF-DDA (w/o BasicNMF) is conducted through 10-fold cross-validation and cold-start test on Gdataset

Test	Metrics	**DNMF-DDA**	DNMF-DDA (w/o KNN)	DNMF-DDA (Single)	DNMF-DDA (w/o BasicNMF)
10-fold CV	AUC	${\textbf {0.954}}$	0.942	0.937	0.951
	AUPR	${\textbf {0.424}}$	0.414	0.383	0.401
	Precision	${\textbf {0.461}}$	0.453	0.427	0.441
Cold-start test	AUC	${\textbf {0.921}}$	0.837	0.850	0.917
	AUPR	${\textbf {0.361}}$	0.336	0.180	0.349
	Precision	${\textbf {0.421}}$	0.409	0.234	0.404

The optimal values are in bold

### Ablation Experiment

Assessing the impact of individual components in the DNMF-DDA model on association prediction involved designing three ablation experiments: DNMF-DDA (w/o KNN), DNMF-DDA (Single), and DNMF-DDA (w/o BasicNMF). Specifically, DNMF-DDA (w/o KNN) indicates that the weighted KNN algorithm is not employed to optimize the association information during the construction of the integrated matrix. DNMF-DDA (Single) denotes that only a single similarity (i.e., ${{\varvec{R}}}_{\textrm{c h e m}}$ and ${{\varvec{D}}}_{\textrm{ph}}$) is used throughout the process for DDA prediction. Meanwhile, DNMF-DDA (w/o BasicNMF) means that the ${{\varvec{W}}}_i$ and ${{\varvec{H}}}_i$ matrices are not pre-trained using NMF but are randomly initialized.

As shown in Table 6, we performed all ablation experiments in Gdataset. DNMF-DDA demonstrates superior performance in all ablation experiments, yielding optimal AUC, AUPR, and precision. The results for DNMF-DDA (w/o KNN) highlight the effectiveness of the KNN algorithm in processing association information through similarity metrics, particularly evident during cold-start tests where KNN significantly enhances the ability to predict unknown associations of new drugs by capturing the intrinsic structural patterns of nodes. The comparison with DNMF-DDA (Single) indicates that this model can extract more comprehensive deep features from multiple information sources, particularly evident in cold-start tests. Complex data often includes significant noise and redundant information. However, DNMF-DDA shows strong capability in identifying and capturing essential deep features. This ability significantly enhances predictive performance, highlighting the model’s potential value in practical applications. Furthermore, the results from DNMF-DDA (w/o BasicNMF) underscore the advantages of BasicNMF to initialize the factor matrices during model solving, which effectively enhances the robustness and quality of the factorization. Overall, in contexts characterized by complex and multidimensional data relationships, DNMF-DDA presents considerable advantages.

### Case Studies

**Fig. 5:**
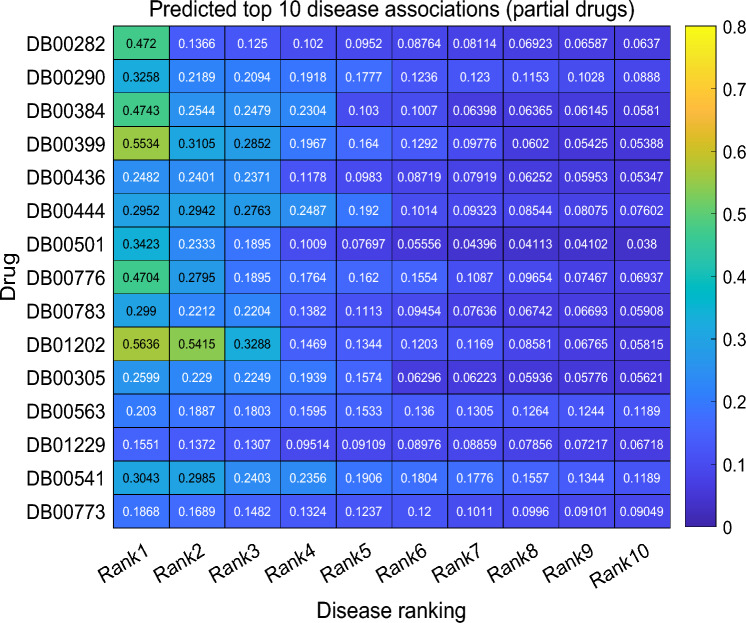
The heatmap of the top 10 disease associations for partial drugs in Gdataset

To identify novel therapeutic applications of approved drugs, we explored the utility of DNMF-DDA through comprehensive case studies. Leveraging all prior information in Gdataset, the DNMF-DDA model was used to discover previously unexplored DDAs. Based on the model’s predictions, we compiled a ranked list of potential indications for each drug using their scores. Figure 5 presents a heatmap of association scores derived from actual predictions, highlighting the top 10 predicted disease associations for a subset of drugs. The complete association score data from Gdataset is available via the GitHub link. In addition, considering the increasing focus on the development of anti-cancer and leukemia therapies in recent years, we selected three widely used anti-cancer drugs (i.e., mitomycin, methotrexate, and paclitaxel) and two drugs for malignant hematologic diseases (i.e., vincristine and etoposide). We analyzed their predicted candidate indications in the CTD database and sought supporting evidence to validate the predicted therapeutic associations.

**Table 7:** The top 10 potential applications for mitomycin, methotrexate, paclitaxel, vincristine, and etoposide

Drug (DrugBank ID)	Top 10 potential diseases (OMIM IDs)
Mitomycin (DB00305)	Osteogenic Sarcoma (259,500); **Colorectal Cancer (114,500)**; **Esophageal Cancer**
	**(133,239)**; **Mismatch Repair Cancer Syndrome 1 (276,300)**; Breast Cancer
	(114,480); Small Cell Cancer of the Lung (182,280); Testicular Germ Cell Tumor
	(273,300); **Myeloma, Multiple (254,500)**; Lymphoma, Hodgkin, Classic (236,000)
	Leukemia, Acute Myeloid (601,626)
Methotrexate (DB00563)	Neuroblastoma, Susceptibility to, 1 (256,700); **Wilms Tumor 1 (194,070)**; Immune
	Thrombocytopenia (188,030); **Myeloma, Multiple (254,500)**; **Lung Cancer**
	**(211,980)**; ** Myelofibrosis (254,450)**; Dermatosis Papulosa Nigra (125,600)
	Thyroid Cancer Nonmedullary 2 (188,470); Kaposi Sarcoma, Susceptibility to (148,000)
	Prostate Cancer (176,807)
Paclitaxel (DB01229)	**Mismatch Repair Cancer Syndrome 1 (276,300)**; Immune Thrombocytopenia
	(188,030); Mycosis Fungoides (254,400); **Testicular Germ Cell Tumor (273,300)**
	Lymphoma, Hodgkin, Classic (236,000); **Neuroblastoma, Susceptibility to, 1**
	**(256,700);** Mccune-Albright Syndrome (174,800); **Diffuse Gastric and Lobular**
	**Breast Cancer Syndrome (137,215)**; Rhabdomyosarcoma 2 (268,220)
	Prostate Cancer (176,807)
Vincristine (DB00541)	**Osteogenic Sarcoma (259,500)**; **Lung Cancer (211,980)**; Bladder Cancer (109,800)
	Thyroid Cancer, Nonmedullary, 2 (188,470); Diffuse Gastric and Lobular Breast
	Cancer Syndrome (137,215); Testicular Germ Cell Tumor (273,300); **Myelofibrosis**
	**(254,450)**; **Leukemia, Chronic Lymphocytic (151,400)**; Glioma Susceptibility 1
	(137,800); Polycythemia Vera (263,300)
Etoposide (DB00773)	Lymphoblastic Leukemia, Acute, with Lymphomatous Features (247,640); **Leukemia,**
	**Chronic Lymphocytic, Susceptibility to, 2 (109,543)**; **Esophageal Cancer**
	**(133,239)**; Reticulum Cell Sarcoma (267,730); Osteogenic Sarcoma (259,500)
	Bladder Cancer (109,800); **Mycosis Fungoides (254,400)**; **Breast Cancer (114,480)**
	Neuroblastoma, Susceptibility to, 1 (256,700); Dohle Bodies and Leukemia (223,350)

The results highlighted in **bold** align with the CTD database findings

As shown in Table 7, the DNMF-DDA algorithm predicts 10 leading candidate indications per drug, with confirmed therapeutic uses emphasized in bold. According to the results, four of the top 10 predicted indications for each drug are verified. For example, Mitomycin (DB00305), a cytotoxic antibiotic that interferes with DNA synthesis and replication in cancer cells, is often prescribed for cancers including esophageal, colorectal, and bladder cancers. As shown in Table 7, three out of its top five predicted indications have already been clinically validated and are used in treatment. However, the rank-first predicted indication, Osteogenic Sarcoma, has yet to be validated as a therapeutic target. This may be due to the rarity of osteosarcoma, which leads to limited omics data and subsequent delays in database validation. Similarly, a comparable pattern is observed with methotrexate. In contrast, key molecular mechanisms of neuroblastoma, such as oncogene amplification and epigenetic regulation, are not fully captured by existing similarity metrics, thereby affecting the establishment of predictive associations [45]. Additionally, some unvalidated predictions require clinical validation and offer potential directions for future research.

This study exhibits the clinical value of DNMF-DDA in predicting new indications for existing drugs, particularly in discovering new applications for anti-cancer treatments. The model’s high predictive accuracy and ability to identify potential research directions highlight its practical utility in drug repositioning.

## Conclusions

In this research, we propose DNMF-DDA, an innovative computational model adept at extracting deep key features to explore prospective DDAs. The model employs an optimization strategy to iteratively refine features, effectively addressing the challenge of predicting indications of new drugs. In both cold-start and 10-fold CV tests, DNMF-DDA demonstrates exceptional predictive performance. It is particularly noteworthy that in the 10-fold CV experiments conducted on Gdataset and Cdataset, DNMF-DDA outperforms existing drug repositioning methods across all evaluation metrics. In the cold-start tests, DNMF-DDA also significantly surpasses other baseline models in terms of AUC. Furthermore, case studies further validate its robustness and practical utility in identifying new applications for established drugs, making DNMF-DDA a promising tool for complex data integration and accurate association prediction. Although the DNMF-DDA method demonstrates superior performance in predicting new drug indications, it still struggles to grasp intricate nonlinear patterns embedded in the data. Furthermore, the high computational complexity arising from multi-layer factorization restricts its application to large-scale datasets. Future research will focus on optimizing the integration of prior information and adopting deep learning techniques to enhance both the precision of drug repositioning and association prediction.

## Data Availability

The source code and datasets for this study are available at https://github.com/YangPhD84/DNMFDDA.
